# Brain responses to negated and affirmative meanings in the auditory modality

**DOI:** 10.3389/fnhum.2023.1079493

**Published:** 2023-01-19

**Authors:** Sara Farshchi, Annika Andersson, Joost van de Weijer, Carita Paradis

**Affiliations:** ^1^Centre for Languages and Literature, Lund University, Lund, Sweden; ^2^Department of Swedish, Linnaeus University, Växjö, Sweden; ^3^Humanities Laboratory, Lund University, Lund, Sweden

**Keywords:** negation, ERP, spoken language processing, sentence comprehension, negated sentences

## Abstract

Negation is frequently used in natural language, yet relatively little is known about its processing. More importantly, what is known regarding the neurophysiological processing of negation is mostly based on results of studies using written stimuli (the word-by-word paradigm). While the results of these studies have suggested processing costs in connection to negation (increased negativities in brain responses), it is difficult to know how this translates into processing of spoken language. We therefore developed an auditory paradigm based on a previous visual study investigating processing of affirmatives, sentential negation (*not*), and prefixal negation (*un*-). The findings of processing costs were replicated but differed in the details. Importantly, the pattern of ERP effects suggested less effortful processing for auditorily presented negated forms (restricted to increased anterior and posterior positivities) in comparison to visually presented negated forms. We suggest that the natural flow of spoken language reduces variability in processing and therefore results in clearer ERP patterns.

## 1. Introduction

Expressions including negation in human communication are rife and have a wide range of different functions such as expressing opposition, rejecting requests, denying information, or mitigating utterances that otherwise would come across as too blunt, assertive, or specific (Giora, 2006; Paradis and Willners, 2006). Negation has been studied in diverse settings, ranging from logic to everyday conversation (Tottie, 1991; Horn, 2010). There is also a rich flora of studies of negation processing that are not entirely in agreement with one another. While some have reported that negation incurs processing costs in most contexts (Just and Carpenter, 1976; Fischler et al., 1983; MacDonald and Just, 1989; Kaup et al., 2006; Ferguson et al., 2008; Lüdtke et al., 2008; Dudschig and Kaup, 2018; Dudschig et al., 2019; Farshchi et al., 2019), others have shown the important role of contextual factors affecting the processing costs (Nieuwland and Kuperberg, 2008; Tian et al., 2010, 2016; Orenes et al., 2014, 2016). More importantly, however, most of the studies using the temporally sensitive measure of event-related potentials (ERPs) to investigate processing of negation are restricted to visual paradigms (but see Herbert and Kübler, 2011 and Herbert and Kissler, 2014). It is possible that the processing of negation as presented visually word by word differs from processing of the same stimuli in a more natural flow of speech. To explore potential effects of mode of presentation, we developed an auditory version of a previously used visual ERP paradigm (Farshchi et al., 2021).

### 1.1. Visual processing of negation

Previous ERP studies of negation in the visual modality have shown a relatively late integration of negation in the comprehension process. This means that ERP effects of negation processing have often shown up in later time-windows, typically 600 ms and onward (Lüdtke et al., 2008) as compared to non-negated information where a typical N400 is observed in earlier time-windows (Fischler et al., 1983). This delay in integration has been argued to be caused by two steps involved in the processing of negated information through the two-step simulation model (Kaup et al., 2006; Lüdtke et al., 2008). The first step involves the simulation of what is expressed in the scope of negation and the second step involves the integration of negation and the simulation of the negated concept.

Other studies emphasizing a dynamic view of negation have highlighted the role of context in processing (Nieuwland and Kuperberg, 2008; Tian et al., 2010, 2016; Orenes et al., 2014, 2016). For instance, contextual cues provided by sentence structure (e.g., simple or cleft) shift the focus of the information and hence affect the representation of the affirmative concept in the integration of negation (Tian et al., 2016). Similarly, Nieuwland and Kuperberg (2008) found that the processing of negation is less effortful in pragmatically plausible contexts than in implausible contexts. More specifically, incongruities in plausible, negated contexts elicited an N400 effect comparable to that elicited by incongruities in affirmative sentences, while this was not the case in implausible contexts.

Revisiting the processing of incongruities in negated and affirmative contexts in a previous ERP study (Farshchi et al., 2021), we investigated the integration of negation in visually presented affirmative and negated sentences such as the ones shown in example (1). The first part of the sentence contained either an affirmative adjective (e.g., *authorized*), a sententially negated adjective (e.g., *not authorized*) or a prefixally negated adjective (e.g., *unauthorized*). Prefixal negation was included because very little is known about the processing of such negated items (Tottie, 1980). The second part of the sentence contained a critical word in the form of one of the members of an antonym pair (e.g., *correct/wrong*), which rendered the whole sentence either congruent or incongruent.

(1) The White House announced that the new Obama biography was authorized/not authorized/unauthorized and the details in the book were correct/wrong in actual fact.

In Farshchi et al. (2021), incongruities in affirmative sentences elicited an N400 reflecting difficulties with the integration of the incongruent word (cf. Kutas and Hillyard, 1980; Fischler et al., 1983) followed by a P600 suggesting a re-analysis of the sentence (cf. Kolk et al., 2003). In sententially negated sentences, however, both congruent and incongruent conditions elicited an equally large N400, suggesting processing costs for the negated forms also in the congruent condition. A difference between incongruent and congruent sententially negated sentences showed up in the form of a later negativity (450–600 ms). This negativity suggests a delayed detection or processing of incongruities due to the complex interpretation of the negated meanings, e.g., *not authorized*, and possibly a memory search for the negated information presented earlier in the sentence. Finally, in prefixally negated sentences, incongruities elicited a sustained anterior negativity, which was interpreted as reflecting taxed working memory processes (cf. Müller et al., 1997; Martín-Loeches et al., 2005) in the retrieval of the prefixally negated meanings, e.g., *unauthorized*. These findings reveal that the processing of sententially negated information was more difficult than that of affirmative information, and the processing of prefixally negated information was even more difficult than the other two forms. The results of Farshchi et al. (2021) do not reflect the two processing steps described by the two-step simulation model. If negation had been processed in two steps, the negator *not* should have been ignored initially, and consequently, the congruent negated condition (*not authorized* with *wrong*) should have elicited a larger N400 compared to the incongruent negated condition (*not authorized* with *correct*). This was, however, not the case. Instead, the results suggest that factors such as the multifunctional meaning potential of *not* and memory retrieval processes have an impact on ease of processing, which highlights the importance of context for the processing of negated meanings.

### 1.2. Auditory processing of negation

As far as we know, the investigation of negated sentences using ERPs in the auditory modality is restricted to two studies (Herbert and Kübler, 2011; Herbert and Kissler, 2014). In Herbert and Kübler (2011), participants were asked to evaluate the truth value of false statements such as *Dogs don’t bark* and true statements such as *Dogs don’t fly*. All sentences were spoken by a female German speaker and an inter-stimulus interval (ISI) of 1500 ms was used between the negation word and the target word, corresponding to a stimulus onset asynchrony of 1810 ms. The ERPs to the target words (*bark/fly*) were recorded. The authors did not find any modulation of the N400 by truth value (e.g., *Dogs don’t fly* vs. *Dogs don’t bark*) (cf. Nieuwland and Kuperberg, 2008) or by semantic relatedness (e.g., semantic relatedness between *dog* and *bark* and semantic unrelatedness between *dog* and *fly* in the statements, respectively) (cf. Fischler et al., 1983; Lüdtke et al., 2008). Instead, false statements elicited a larger frontal negativity (300–600 ms) followed by a larger parietal positivity (600–1000 ms). We argue that the lack of an N400 effect in response to semantic relatedness may have been caused by the relatively long ISI of 1500 ms between the negator (*n’t*) and the target word (*bark/fly*), which may have allowed for processing and integration of negation. Note, however, that if negation had indeed been integrated, the N400 ought to have been modulated in the congruent condition (i.e., increased N400 for *bark* as a dog indeed barks), which it did not. The length of the ISI could have affected the pattern of ERP effects as this ISI is significantly longer than the typical ISIs used in ERP paradigms.

In the second study, Herbert and Kissler (2014) used the same sentences as Herbert and Kübler (2011) but with a shorter ISI between the negator and the target word (approximately 500 ms). The experiment consisted of a passive listening task followed by an active truth evaluation task with repeated presentations of the sentences. The question was whether the first task would facilitate the processing of negated sentences in the second task (resulting in enhanced N400 and late positive potential), or would lead to the same effects attenuated due to the repetition of the sentences. In the second task, half of the participants performed the truth evaluation mentally and the other half did the evaluation through button presses. The overall effects were larger in the active task than in the passive task, suggesting that active evaluation of sentences requires more resources than passive evaluation. Additionally, and in line with the results of the first study, Herbert and Kübler (2011) found a larger late positive potential for incongruent negated sentences compared to congruent negated sentences in the passive task.

Despite the valuable findings of these two studies, we argue that the presentations were somewhat unnatural: either the negated forms were presented with long ISIs or stimulus sentences were repeated, which could have affected processing. Also, no affirmative counterpart was included in the design of these studies that would allow for a comparison between the processing of negated and non-negated information. This calls for further exploration of processing negation in the auditory modality, which is what we do in the present study.

## 2. The present study

In the present study we are concerned with (i) whether there is a cost in auditory processing of negated sentences compared to affirmative sentences, and (ii) whether there are any differences between auditory processing and the previously reported visual processing of the sentences (Farshchi et al., 2021). To this end, we use identical stimulus sentences (Example 1) as in Farshchi et al. (2021) but present them auditorily rather than visually.

The auditory modality is a more immediate and natural way of processing language, and thus it is expected that spoken sentences be integrated more easily compared to visually presented sentences in a word-by-word paradigm. Previous ERP studies of auditory language processing have shown different ERP patterns between the two modalities (McCallum et al., 1984; Holcomb and Neville, 1990, 1991). Some have shown ERP effects showing up in earlier time-windows (Holcomb and Neville, 1990). We expect to replicate the finding of an N400, and possibly a P600 effect in response to the incongruities in affirmative sentences. Moreover, we expect the previously reported unclear pattern of ERP effects associated with the visual processing of incongruities in prefixally and sententially negated sentences in Farshchi et al. (2021) (i.e., sustained anterior negativity and delayed negativity) to show clearer patterns because the auditory processing of speech is more common than visual processing. More specifically, if negation is integrated, we expect incongruities in sententially negated contexts to elicit either a larger N400 effect (in line with Nieuwland and Kuperberg, 2008) or a larger P600 effect [in line with Herbert and Kübler (2011) and Herbert and Kissler (2014)], or possibly both (N400-P600 effect). Similarly, in regard to the rarely researched prefixally negated forms, we expect clearer and more established processing patterns than when the sentences were presented visually word-by-word. For the behavioral data, we expect to see lower accuracy rates and longer response times for the incongruent conditions compared to the congruent conditions in all three sentence types. In line with Farshchi et al. (2021), we expect to see lower accuracy rates for prefixal negation compared to the affirmative and sentential negation conditions.

## 3. Methods

### 3.1. Participants

Thirty-two right-handed native speakers of English (21 females; mean age = 24.8 years, range = 20–33 years) participated in the study with the following countries of origin: US (12), UK (10), Canada (3), Australia (3), South Africa (2), Ireland (1) and New Zealand (1). All participants listed English as their native language. Six participants listed an additional language as their second native language. All participants provided written consent prior to the experiment and received a cinema voucher as compensation. None reported any neurological disorders or hearing problems.

### 3.2. Stimuli

The stimulus sentences from Farshchi et al. (2021) were read aloud by a female native speaker of British English and recorded specifically for the experiment. One item from the previous stimulus set was deemed unnatural and removed. The total number of sentences was 306 sentences (17 adjective sets × 3 sentence frames × 6 conditions) presented in three lists of 102 sentences each so that each participant listened to only two conditions of the same sentence frame. Sentence frame refers to one of the three sentential contexts created for each adjective set. Condition refers to the combination of negation (affirmative/prefixal negation/sentential negation) and congruency (congruent/incongruent). See Supplementary Appendix A for a complete list of stimulus sentences. The final lists were pseudo-randomized in that the sentences from the same frame were separated by at least two or more other frames.

To ensure that the ERP effects were elicited by the manipulations only, identical primes and critical words were used. The stimulus sentences were spliced into four different parts (Table 1) with the use of Audacity (Audacity team, 2019) and Praat speech editing software (Boersma and Weenink, 2019). The sentence parts were combined into one audio file with breaks of 9 and 11 ms, respectively before the critical words (parts 2 and 4 in Table 1). These intervals were chosen based on the average pause length between the offset of the preceding word and the onset of the target word in the original recordings. The average duration of the sentences was 7.13 s (SD = 0.96). The EEG was time-locked to the onset of the critical word (part 4 in Table 1), which was identified using Praat. PsychoPy software program (Peirce et al., 2019) was used for the presentation of the experiment.

**TABLE 1:** Splicing scheme of the stimuli in one sentence frame for the adjective set “authorized/unauthorized/not authorized”.

Part 1	Part 2	Part 3	Part 4
	authorized		correct in actual fact
The new Obama biography was	unauthorized	therefore the details in the book were	
	not authorized		wrong in actual fact

EEG was time-locked to the onset of part 4.

### 3.3. Procedure

Participants were informed about the procedure, signed a consent form, and were seated at a 110 cm distance from a monitor with an electrode cap on their head. Participants were instructed to focus on a cross in the middle of the screen while listening to sentences presented through loudspeakers placed on either side of the screen. After each trial, three question marks appeared on the screen prompting participants to answer “yes” or “no” to the question “Did the sentence make sense, logically?” by pressing a green or red button on a keyboard. The placement of the buttons was counter-balanced across participants. Response accuracy, response time, and continuous EEG were recorded. There was no official pause or break although participants were told they could take a short break if needed. The task lasted approximately 40 min.

### 3.4. EEG recordings and processing

EEG recording and processing criteria were identical to those presented in Farshchi et al. (2021). Continuous EEG data was recorded at a 500-Hz sampling rate from 30 scalp electrodes (NeuroScan Easycap), 2 mastoid electrodes and 4 facial electrodes for recordings of EOG. From a total of 3,264 trials (102 items by 32 participants), 3% of the data was rejected with similar number of trials in each condition (519–534).

### 3.5. Data analysis

The procedure for building statistical models was identical to that adopted in Farshchi et al. (2021). In the analyses of both behavioral and ERP data, sentence type (affirmative, sentential negation, and prefixal negation) and congruency (congruent and incongruent) were used as predictors. In all the analyses, affirmative and congruent conditions were coded as the reference level. Subject and item information were included as random effects. Item information was only available for behavioral data.

For ERPs, the mean amplitude in three time-windows was analyzed in order to capture three responses observed in previous auditory studies (Herbert and Kübler, 2011; Herbert and Kissler, 2014): the N400 (450–650 ms), the P600 (650–800 ms), and a late positive potential (LPP, 800–1000 ms). Six separate analyses were performed for the three time-windows over the anterior region (electrode sites F3/4, FZ, FC3/4, FCZ, C3/4, and CZ) and the posterior region (CP3/4, CPZ, P3/4, PZ, O1/2, and OZ) as differences in topographical distributions have previously been observed between the visual and auditory modalities (e.g., McCallum et al., 1984; Holcomb and Neville, 1990, 1991). *Post hoc* pairwise comparisons were performed, and z-scores were adjusted accordingly. Supplementary Appendix B contains more details about the analyses.

## 4. Results

### 4.1. Accuracy rates and response times

The proportions of accurate responses and mean response times within the congruent and incongruent conditions for each sentence type are listed in Table 2. In the analysis of the accuracy rates, the model with the strongest predictive accuracy included a significant interaction between sentence type and congruency. The output of the model revealed lower accuracy rates for both prefixal negation (β = –0.88, SE = 0.18, *z* = –4.67, *p* < 0.001) and sentential negation (β = –0.70, SE = 0.19, *z* = –3.66, *p* < 0.001) compared to affirmative forms within the congruent condition. Lower accuracy rates were found for the incongruent than the congruent condition in affirmative sentences (β = *–0.84*, SE = *0.20, z* = –4.08, *p* < 0.001) but a significant interaction effect between both negation types and congruency revealed that this direction of effects was not observed for prefixal negation (β = 1.19, SE = 0.25, *z* = 4.68, *p* < 0.001) or sentential negation (β = 0.80, SE = 0.25, *z* = 3.16, *p* < 0.01). No other congruency effects were found for the two negation types, nor were there any significant differences between the two negation types.

**TABLE 2:** Proportions of accurate responses and average response times for the three sentence types.

Sentence type	Proportion accurate responses		Average response time (sec)	
	Congruent	Incongruent	Congruent	Incongruent
Affirmative	0.91	0.83	0.94	1.11
Sentential negation	0.84	0.84	1.13	1.16
Prefixal negation	0.81	0.86	1.11	1.22

For the response time analysis, there were neither significant interaction effects nor significant effects of sentence type. However, the main effect of congruency was significant suggesting that the responses in the incongruent condition were slower than those in the congruent condition (β = 0.13, SE = 0.04, *t* = 2.17, *p* < 0.05).

### 4.2. ERPs

**FIGURE 1:**
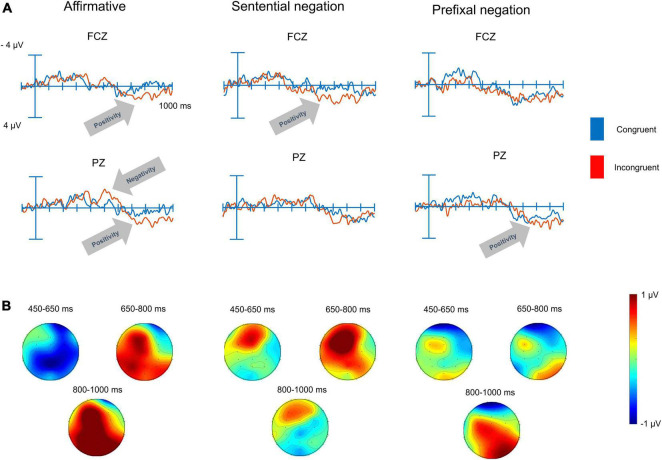
Grand averages and topographic maps of the ERP responses to auditory processing of affirmative and negated sentences. **(A)** The grand averages for the congruent and incongruent conditions in two representative electrodes from the anterior (FCZ) and posterior regions (PZ). **(B)** The topographic maps of the difference between the incongruent and the congruent conditions for affirmative sentences, sententially negated and prefixally negated sentences (see Supplementary Appendix C for a plot of all 18 electrodes for the three conditions).

As seen in Figure 1, incongruities in affirmative sentences elicited an N400 effect over the posterior region. This N400 effect was followed by a P600 effect over the same region and continued through the third time-window (1000 ms) over both regions. For sententially negated sentences, no N400 effect was observed for the incongruities but a larger positivity for incongruities in the P600 time-window over the anterior region. For prefixally negated sentences, the effect of incongruity was restricted to a larger positivity in the LPP time-window, and this effect approached significance over the posterior region.

In all six analyses of the ERP data, a significant interaction between the two predictors, sentence type and congruency, was observed. We pursued the interactions from each analysis with three contrasts between the congruent and incongruent conditions within each sentence type. The results of these contrasts are shown in Table 3 and discussed below.

**TABLE 3:** *Post-hoc* contrasts (general linear hypothesis tests) between the congruent and incongruent conditions in the three sentence types over the two regions of interest.

	Posterior	Anterior
**N400**		
Affirmative	β = –0.77, SE = 0.32, *z* = –2.14*	NS
Sentential negation	NS	NS
Prefixal negation	NS	NS
**P600**		
Affirmative	β = 0.71, SE = 0.36, *z* = 1.97^#^	NS
Sentential negation	NS	β = 0.90, SE = 0.33, *z* = 2.70*
Prefixal negation	NS	NS
**LPP**		
Affirmative	β = 1.15, SE = 0.38, *z* = 2.98**	β = 0.91, SE = 0.36, *z* = 2.48*
Sentential negation	NS	NS
Prefixal negation	β = 0.73, SE = 0.38, *z* = 1.90^#^	NS

β represents the estimated mean difference amplitude in microvolts between the congruent and incongruent conditions in each affirmative, sentential negation and prefixal negation sentence type. SE represents the standard error. NS signifies non-significant effects. Significant and marginally significant z scores are marked as follows: ^#^0.10–0.05; *p < 0.05; **p < 0.01.

## 5. Discussion

This study has investigated how negation in auditorily presented sentences is processed. More specifically, the aim was to explore if there are differences in the auditory processing of negated meanings and that of affirmative ones. Previous studies investigating negated meanings have either been limited to visual word-by- word presentations (e.g., Lüdtke et al., 2008; Nieuwland and Kuperberg, 2008), or to the auditory processing of negated forms without including an affirmative counterpart (e.g., Herbert and Kübler, 2011; Herbert and Kissler, 2014). Using the same stimuli as in our previous visual study (Farshchi et al., 2021), we zoomed in on whether auditory processing of negation incurs a cost for processing in the same way as visual processing does.

Overall, the patterns in the behavioral and neurophysiological responses to the auditory stimuli indicate that the affirmative form was easiest to process, followed by sentential negation and prefixal negation in that order. Incongruities in affirmative sentences yielded a large N400 followed by a large P600. This is in line with previous findings of violations of semantic expectancy in affirmative sentences where a large N400 response suggests a cost of processing and integration of an incongruent word into the semantic context (Kutas and Hillyard, 1980; Fischler et al., 1983; Kolk et al., 2003; Farshchi et al., 2021), while a large P600 response suggests a re-evaluation of the whole sentence (Kolk et al., 2003; van Herten et al., 2005; Bornkessel-Schlesewsky and Schlesewsky, 2008).

Having thus established the patterns in the affirmative (baseline) condition, we proceed to the processing of the two negated forms, where we found no N400 effect in response to the incongruities in either of the two negated conditions (sentential and prefixal). This finding is in line with that of the previous auditory studies (Herbert and Kübler, 2011; Herbert and Kissler, 2014) and suggests that the processing of incongruities in negated contexts is more complicated than processing incongruities in affirmative contexts. In contrast to these previous studies, however, we found an anterior positivity in the P600 time-window for incongruities in sententially negated sentences. The anterior distribution of the positivity rather than a replication of a parietal positivity could possibly be related to the nature of the stimuli and the task in the current study. Indeed, an anterior P600 has been reported previously for congruent plausible but unpredictable sentence completions in affirmative contexts (see Van Petten and Luka, 2012). One of the functions of the negator *not* is that it creates a conceptual space that potentially can cover anything, not only what is in the scope of the negated items. Because of this, it can be argued that the incongruent endings in negated sentences were processed as unpredictable but nevertheless acceptable endings rather than clear-cut incongruities (see Farshchi et al., 2021 for a detailed discussion).

For prefixally negated sentences, incongruities elicited a posterior positivity in the LPP time-window. This positivity, which has a longer latency than the positivities elicited in sententially negated and affirmative sentences, can be a temporal indication of the point in time when the participants detected the incongruity and attempted to re-evaluate the context, hence suggesting that it took longer for participants to identify and process the incongruities in prefixally negated sentences compared to the incongruities in the other two types of sentences. The combination of the lower accuracy rates compared to the affirmative condition and the LPP is evidence in favor of prefixal negation being the most difficult condition to process. We argue that this difficulty may have been caused by the less natural use of prefixally negated forms at the beginning of sentences. Prefixally negated forms are highly assertive and may come across as unnatural when used at the beginning of the sentence without a lead-in (for a discussion, see Farshchi et al., 2021).

Our results do not provide support for the two views on the processing of negation. Firstly, we did not find any evidence for the initial simulation step in the two-step model (Fischler et al., 1983; Lüdtke et al., 2008). The N400 was not larger for the congruent negated condition than the incongruent negated condition, which would have been expected if negation had been ignored in the first step as is predicted by the two-step model. Secondly, we did not find support for a larger N400 elicited by incongruities as Nieuwland and Kuperberg (2008) did. The lack of a larger N400 for incongruities in our study may have been incurred by the nature of the incongruities in sententially negated sentences, which were not as strong as those in the affirmative sentences. In other words, the contexts created by negation were not as semantically predictable as those by affirmative forms and hence, the violations of semantic expectancy in the negated sentences did not give rise to an N400 effect. We do, however, align our findings with a dynamic view of negation advocating the role of contextual factors and in particular, the type of modality of sensory input, in the processing of negated meanings. This will be further elaborated on in the next section.

### 5.1. Auditory vs. visual processing of negation

When comparing the findings from the auditory processing with those from the visual processing of the same sentences (Farshchi et al., 2021), the N400–P600 response for the incongruities in affirmative sentences in the auditory modality was replicated, which signals that the affirmative sentences were easiest to process and rightly served as the baseline.

For sententially negated sentences, the ERP patterns differed between the two modalities. In the visual modality, incongruities elicited a larger centro-parietal negativity in the P600 time-window, which was taken to reflect delayed and costly processing of the negated sentences. In the auditory modality, incongruities in the sententially negated sentences instead elicited a larger positivity (650–800 ms) over the anterior region. We argued earlier that this positivity could indicate the weak nature of the incongruities in negated contexts where the incongruent endings might have been taken as felicitous sentence completions.

In both modalities, prefixal negation is the most difficult condition to process based on behavioral and ERP patterns. In the visual processing of these forms (Farshchi et al., 2021), incongruities elicited a sustained anterior negativity expanding over both time-windows (300–600 ms), which was taken to reflect higher cognitive effort and working memory load (Müller et al., 1997; Martín-Loeches et al., 2005). In the auditory processing of these sentences, instead, a larger posterior positivity (600–1000 ms) was found for incongruities. This points to a delayed and cognitively more demanding process in sentence comprehension and integration (Kolk et al., 2003; van Herten et al., 2005; Bornkessel-Schlesewsky and Schlesewsky, 2008). Together, these ERP effects reflect a greater difficulty in processing prefixally negated contexts compared to the other conditions.

Overall, the results indicate that there are many similarities between the two modalities, at least in terms of the overall difficulty in processing between the conditions. It may be argued that the effects found in the auditory processing of negated sentences (late positivities) are easier to interpret and align with previous findings as they are more well-established and more commonly found in studies of violations of semantic expectancy (Kolk et al., 2003; van Herten et al., 2005; Bornkessel-Schlesewsky and Schlesewsky, 2008). Accordingly, it can be argued that the auditory processing of negated sentences is easier than their visual word-by-word processing as the ERP effects in the visual modality (sustained anterior and centro-parietal negativities) reflected a higher working memory and cognitive load, which may be factors that have masked the actual cognitive processes behind the comprehension of negated meanings.

## 6. Conclusion

In this study we have targeted the auditory processing of negated sentences and compared it to that of affirmative sentences. The results show that the sententially and prefixally negated sentences were more difficult to process than the affirmative sentences. Incongruities in affirmative sentences were easily detected as indicated by an N400 and a P600 effect, but that was not the case for the negated sentences with *not* (anterior positivity) and *un*-prefixed sentences (posterior positivity).

The findings suggest that the auditory processing of negated sentences is easier than visual word-by-word processing which is a method typically adopted in visual ERP studies. This facilitation is suggested by the elicitation of more established effects in response to semantic incongruities in negated sentences (late positivities), possibly because the processing of spoken language is omnipresent and used by all speakers all the time. In that sense it is more natural than the processing of written language and in particular so when the writing is encountered through a word-by-word presentation paradigm. This conclusion needs to be consolidated in future studies in which the processing of affirmative and negated sentences in the auditory and visual modalities is compared in a single experimental setup. Hopefully, this investigation will stimulate more comparative investigations of the processing of additional discursive phenomena across modalities for a better understanding of spoken language processing and what may be the reason for why it is relatively effortless.

## Data availability statement

The raw data supporting the conclusions of this article will be made available by the authors, without undue reservation.

## Ethics statement

The studies involving human participants were reviewed and approved by the Swedish Ethical Review Authority (Etikprövningsnämnden I Lund): 2016 996-2. The patients/participants provided their written informed consent to participate in this study.

## Author contributions

SF was the lead researcher of the team for the whole enterprise and was responsible for the creation and design of the study and stimuli, carrying out the experiment, performing the analyses, and led the writing-up of the manuscript. AA contributed with her expertise in the ERP methodology for language processing. JW contributed with his statistical expertise. CP contributed with linguistic expertise in particular on negation. AA, JW, and CP read and made comments and concrete textual suggestions. All authors contributed to the article and approved the submitted version.
