# Supplementary material for: Brain responses to negated and affirmative meanings in the auditory modality
Source: Front Hum Neurosci. 2023 Jan 19;17:1079493. doi: 10.3389/fnhum.2023.1079493 (PMC9892462; doi:10.3389/fnhum.2023.1079493)
Supplement: Supplementary file 1 [file Data_Sheet_1.pdf]

# Appendix A. List of all stimuli sentences and the log frequencies of items and critical words (within parentheses)

For each adjective set, three sentence frames were created. In each sentence, ADJ indicates one of the three adjectives in the adjective set. The underlined words are the critical words that were the target words for the ERP analysis and were kept constant across the three sentence frames. The log frequencies have been given for lexical items, that is, affirmative and prefixally negated forms and not for sententially negated expressions.

---

*intentional (8.04)*
*unintentional (6.69)*
*not intentional*

*guilty (10.14)*
*innocent (9.66)*

The fire was ADJ so the jury found him guilty/innocent in court

The oil spill was ADJ so the company was guilty/innocent of misconduct

The environmental damage was ADJ so the company was found guilty/innocent of wrongdoing

---

*paid (8.23)*
*unpaid (7.68)*
*not paid*

*allowed (10.95)*
*prohibited (8.30)*

During parental leave male employees were ADJ because paid leave was allowed/prohibited by the law

All her speeding tickets were ADJ therefore the renewal of her license was allowed/prohibited by the court

The internship at the hospital was ADJ therefore taking extra shifts was allowed/prohibited for interns

---

*authorized (7.20)*
*unauthorized (7.57)*
*not authorized*

*correct (10.47)*
*wrong (11.44)*

The new Obama biography was ADJ therefore the details in the book were correct/wrong in actual fact

Her access to the food storage was ADJ therefore opening it for her friends to get food was correct/wrong according to her

The information that she disclosed was ADJ therefore the board agreed that her decision was correct/wrong in taking

| | |
|---|---|
| *lawful (7.14)*<br>*unlawful (7.32)*<br>*not lawful* | *resumed (8.10)*<br>*ceased (8.12)* |

The police announced that the rally was ADJ so the protest resumed/ceased immediately

The house search was ADJ therefore the police hunt for evidence resumed/ceased directly

The court decided that the drug-test was ADJ hence the medical experiment resumed/ceased at once

| | |
|---|---|
| *defeated (7.68)*<br>*undefeated (7.03)*<br>*not defeated* | *elimination (8.16)*<br>*qualification (7.15)* |

In the semi-finals Brazil was ADJ and this led to their elimination from/qualification for the final

The tennis player was ADJ and this resulted in his elimination from/qualification for the tournament

At the horse show the current champion was ADJ and this resulted in his elimination from/qualification for the Olympics

| | |
|---|---|
| *restricted (7.87)*<br>*unrestricted (7.09)*<br>*not restricted* | *improved (9.99)*<br>*reduced (10.15)* |

Employees' access to social-media websites was ADJ so their efficiency was improved/reduced significantly

Access to clean water was ADJ and this led to women's health conditions being improved/reduced over time

His access to the team's resources was ADJ which resulted in his performance being improved/reduced significantly

| | |
|---|---|
| *impressed (6.64)*<br>*unimpressed (6.42)*<br>*not impressed* | *increased (10.91)*<br>*decreased (8.86)* |

The critics were ADJ by the show so the ticket sales increased/decreased after the reviews

After her performance the coaches were ADJ and their expectations increased/decreased considerably

After the debate people were ADJ therefore the number of supporters increased/decreased in size

| | |
|---|---|
| *recognized (7.13)*<br>*unrecognized (6.55)*<br>*not recognized* | *appreciated (8.62)*<br>*neglected (8.50)* |

Before his death the great poet was ADJ so his major works were appreciated/neglected during his lifetime

His expertise in the field was ADJ and his efforts were appreciated/neglected by the scientific community

After many years of struggle his talent was ADJ and his efforts were appreciated/neglected by the music industry

| | |
|---|---|
| *anticipated (7.92)*<br>*unanticipated (6.68)*<br>*not anticipated* | *mentioned (10.39)*<br>*ignored (9.59)* |

The side-effects of the drug were ADJ so the consent form mentioned/ignored them in every case

The storm was ADJ so grey skies were mentioned/ignored in the weather forecast

The financial crisis was ADJ so its early signs were mentioned/ignored by analysts

| | |
|---|---|
| *licensed (7.85)*<br>*unlicensed (6.35)*<br>*not licensed* | *performed (10.06)*<br>*skipped (7.82)* |

The gun dealer was ADJ therefore the criminal background check was performed/skipped prior to the sale

The surgeon was ADJ therefore all the routines before the operation were performed/skipped as he ordered

The pilot was ADJ therefore the safety procedures were performed/skipped before takeoff

| | |
|---|---|
| *conditional (7.19)*<br>*unconditional (7.38)*<br>*not conditional* | *pressured (7.78)*<br>*relaxed (9.02)* |

The university's scholarships are ADJ so students feel pressured/relaxed during exams

Admission on the national team is ADJ so each gymnast feels pressured/relaxed about her performance

Model jobs are ADJ therefore models feel pressured/relaxed about their diet

---

| | |
|---|---|
| *readable (6.50)* | *maintained (9.44)* |
| *unreadable (6.26)* | *damaged (9.20)* |
| *not readable* | |

The warning label on the pack of cigarettes was ADJ so the company’s reputation was maintained/damaged in the marketplace

Her final essay was ADJ so her good standing was maintained/damaged in class

The ancient script was ADJ therefore this cultural treasure was considered maintained/damaged by the committee

---

| | |
|---|---|
| *employed (6.10)* | *negligible (7.27)* |
| *unemployed (8.49)* | *noteworthy (7.78)* |
| *not employed* | |

Most women in the community were ADJ so gender differences in employment were negligible/noteworthy at the time

Most immigrants are ADJ so integration problems are negligible/noteworthy in the country

The majority of graduates are ADJ so the effects of the recent recession are negligible/noteworthy for university students

---

| | |
|---|---|
| *even (7.34)* | *small (12.29)* |
| *uneven (8.03)* | *large (11.96)* |
| *not even* | |

The distribution of wealth was ADJ and the gap between social classes was small/large as a result

The sidewalk was ADJ so the number of pedestrian accidents was small/large during winter

The economic growth in the region was ADJ so the political disagreements were small/large among the countries

---

| | |
|---|---|
| *insured (6.16)* | *recovered (9.09)* |
| *uninsured (7.84)* | *lost (11.71)* |
| *not insured* | |

His investment was ADJ so after bankruptcy his money was recovered/lost down to the last cent

His house was ADJ so after the burglary his valuables were recovered/lost entirely

The cancer patient was ADJ therefore after the expensive treatment his life savings were recovered/lost entirely

---

| | |
|---|---|
| *decided (5.84)* | *high (12.46)* |
| *undecided (7.63)* | *low (11.41)* |
| *not decided* | |

By the age thirty her future was ADJ so her chances of a comfortable retirement were high/low on paper

The audience at the contest were ADJ so her chances of winning their votes were high/low at this point

After the court recess the jury was ADJ so the chances of a retrial high/low as expected

| | |
|---|---|
| *forgettable (5.98)* | *talented (9.12)* |
| *unforgettable (7.23)* | *mediocre (7.51)* |
| *not forgettable* | |

The characters in the movie were ADJ so the director was known to be talented/mediocre by the film industry

Since most of his songs were ADJ he was known to be talented/mediocre as an artist

The performances were ADJ as the actors were talented/mediocre in the show

| | |
|---|---|
| *resolved (4.90)* | *ended (10.57)* |
| *unresolved (7.54)* | *continued (10.93)* |
| *not resolved* | |

The chairman announced that the budget issue was ADJ so this discussion ended/continued accordingly

He felt that his childhood emotional conflicts were ADJ so the therapy sessions ended/continued subsequently

Under his leadership the economic problems were ADJ so his managerial role ended/continued in the company

# Appendix B

The eight tables below present the specifications for the best models in the analyses of accuracy rates, response times and ERP amplitudes. The predictors used in creating the models for the behavioural data include: Sent(ence) type, Congruency, Subject and Item. Predictors used in creating the models for the ERP data include: Sent(ence) type, Congruency, Subject and Channel.

*Random effects and significant fixed effects for accuracy rate*

| Model for accuracy (glmer):<br>Accuracy ~ Sent type:Congruency + Sent type + Congruency + (1 \| Subject) + (Congruency \| Item) | | | |
|---|---|---|---|
| Random effects | **Variable** | **Variance** | **SD** |
| *Subject* | (intercept) | 0.12 | 0.35 |
| *Item* | (intercept)* | 0.56 | 0.75 |
| | Congruency-incong | 0.06 | 0.25 |
| Fixed effects | | | |
| | **Estimate** | **Std. Error** | **Z value** |
| Intercept** | 2.55 | 0.25 | 10.23 |
| Sent type-not | -0.70 | 0.19 | -3.66 |
| Sent type-un | -0.88 | 0.18 | -4.67 |
| Congruency-incong | -0.84 | 0.20 | -4.08 |
| Sent type-not: Congruency-incong | 0.80 | 0.25 | 3.16 |
| Sent type-un: Congruency-incong | 1.19 | 0.25 | 4.68 |

*Intercept: congruent condition
** Intercept: affirmative congruent condition

*Random effects and significant fixed effects for response times*

| Model for response time (lmer): Response time ~ Sent type + Congruency + (1 \| Subject) + (Sent type \| Item) | | | |
|---|---|---|---|
| Random effects | **Variable** | **Variance** | **SD** |
| *Subject* | (intercept) | 0.24 | 0.49 |
| *Item* | (intercept)* | 0.06 | 0.26 |
| | Sent type-not | 0.12 | 0.35 |
| | Sent type-un | 0.05 | 0.22 |
| Fixed effects | | | |
| | **Estimate** | **Std. Error** | **t value** |
| Intercept** | 1.08 | 0.11 | 9.14 |
| Sent type-not | 0.11 | 0.10 | 1.14 |
| Sent type-un | 0.13 | 0.07 | 1.72 |
| Congruency-incong | -0.10 | 0.04 | -2.17 |

*Intercept: affirmative condition
**Intercept: affirmative congruent condition
