# Supplementary material for: Brain responses to negated and affirmative meanings in the auditory modality
Source: Front Hum Neurosci. 2023 Jan 19;17:1079493. doi: 10.3389/fnhum.2023.1079493 (PMC9892462; doi:10.3389/fnhum.2023.1079493)
Supplement: Supplementary file 2 [file Data_Sheet_2.pdf]

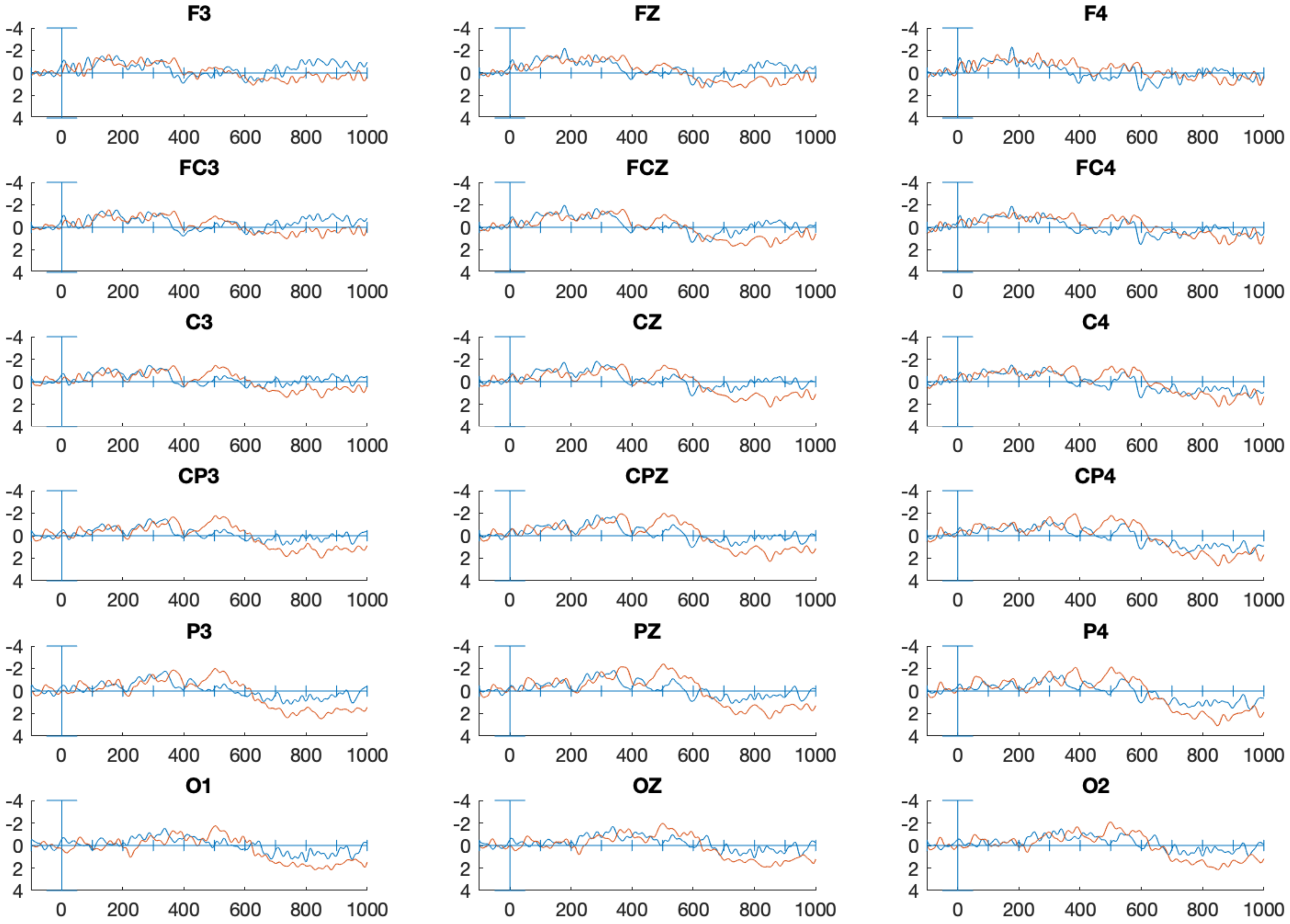

Plot of all 18 electrodes for incongruent and congruent conditions in affirmative sentences. Note that the response to the congruent sentences is shown in blue and the response to the incongruent sentences in red.
